# Differential resilience of Amazonian otters along the Rio Negro in the aftermath of the 20^th^ century international fur trade

**DOI:** 10.1371/journal.pone.0193984

**Published:** 2018-03-30

**Authors:** Natalia C. Pimenta, André P. Antunes, Adrian A. Barnett, Valêncio W. Macedo, Glenn H. Shepard

**Affiliations:** 1 Amazon Mammal Research Group, National Institute for Amazonian Research, Manaus, Amazonas, Brazil; 2 Institutional Capacity Building Program/MCTIC, Department of Anthropology, Emílio Goeldi Museum, Belém, Pará, Brazil; 3 Department of Ecology, National Institute for Amazonian Research, Manaus, Amazonas, Brazil; 4 Comunidade Urumutum Lago, rio Ayari, Escola Indígena Baniwa e Coripaco Pamáali, São Gabriel da Cachoeira, Amazonas, Brazil; 5 Department of Anthropology, Emilio Goeldi Museum, Belém, Pará, Brazil; University of Kwazulu-Natal, SOUTH AFRICA

## Abstract

Commercial hunting for the international trade in animal hides in the 20^th^ century decimated many populations of aquatic wildlife in Amazonia. However, impacts varied significantly between different species and regions, depending upon hunting intensity, accessibility of habitat, and the inherent resilience of various species and their habitats. We investigated the differential responses of two Amazonian Mustelid species, the neotropical otter and giant otter, to commercial hunting pressure along the upper Rio Negro in Brazil, and examined historical factors that influenced spatial and temporal variation in commercial exploitation. We analyzed previously unanalyzed data from historical records of hide shipments to track changes in hide sales and prices for the two species in the late 20^th^ century. We also gathered oral histories from older Baniwa people who had witnessed or participated in commercial otter hunting. These complimentary data sources reveal how intrinsic biological and social characteristics of the two otter species interacted with market forces and regional history. Whereas giant otter populations were driven to local or regional extinction during the late 20^th^ century by commercial hunting, neotropical otters persisted. In recent decades, giant otter populations have returned to some parts of the upper Rio Negro, a development which local people welcome as part of a generalized recovery of the ecosystems in their territory as a result of the banning of animal pelt exports and indigenous land demarcation. This paper expands the scope of the field historical ecology and reflects on the role of local knowledge in biodiversity conservation.

## Introduction

Resource use patterns throughout Amazonia have been shaped by the enduring legacy of the Rubber Boom at the turn of the 20^th^ century (1895–1912). To supply international demand for wild rubber, merchants from Amazonian cities sent commercial fleets into hinterlands to exploit rubber tappers in a system of debt peonage known in Brazil as *aviamento* [1]. When wild rubber prices collapsed in 1912 due to the success of rubber plantations in Malaysia, some rubber merchants survived by using their existing fleets and routes to exploit alternative forest products, especially wild animal hides [2]. The international trade in Amazonian hides reached its first peak during WWII, when the capture of Malaysian rubber plantations by the Japanese led to a brief second Rubber Boom in 1942–1945 [3]. Fueled by demand from US and European markets, at least 23 million animals were hunted in the western Amazon for their hides between 1904 and 1969 [2,4]. A series of laws [5,6] attempted to regulate this trade, until the complete prohibition of commercial hunting in Brazil in 1967 [7]. However, an extension of the deadline for liquidating “stockpiled” hides and skins resulted in the maintenance of illegal commercial hunting until 1974 [8]. Only after Brazil’s adherence to the 1975 Convention on the International Trade in Endangered Species (CITES), which banned the otter skin trade completely, was there a significant reduction in the commercial demand and, consequently, in the legal hunting of wild animals [9].

Commercial hunting had especially severe impacts on large aquatic species, leading to the widespread collapse of populations of giant otter (*Pteronura brasiliensis*), black caiman (*Melanosuchus niger*) and manatee (*Trichechus inunguis*) across the Amazon Basin [10]. The relative ease of access to riverine habitats and the concentration of human population along the major waterways contributed to this "Empty River" scenario [4,10]. The neotropical otter and the giant otter, both semi-aquatic Mustelide carnivores, were hunted commercially for their pelts. In the late 1960s, a single neotropical otter skin would sell on the international market for US$ 175.00 and a giant otter skin for US$ 440.00 (calculated at 2015 values). From 1904 to 1969, at least 390,000 giant otters, and 370,000 neotropical otters, were killed for their pelts in the central-western Brazilian Amazon [4]. Otter populations declined severely in areas wherever they were hunted commercially [11,12]. As a direct result of this commercial hunting, the giant otter, originally distributed widely from Venezuela to Argentina, is now considered extinct in much of its historic range [12,13].

Each Amazonia region has its own unique historical trajectory with regard to subsistence and commercial hunting [4], reflecting local factors such as changes in hunting intensity through space and time, market access, socioeconomic and cultural variation and environmental carrying capacity [4,14,15]. Analyzing the response of animal populations to hunting pressure helps refine concepts relating to species resilience, extinction and other large-scale ecological processes [4,15]. To date, most studies investigating the impact of hunting on Neotropical wildlife have been restricted to terrestrial fauna [16–18]. Also, they are generally based on a central-place foraging model [19–23], which can be inappropriate when considering the differential accessibility of terrestrial environments vs. wetlands in Amazonia and distinctive patterns of mobility in such habitats.

The study of past ecological events requires the integration of multiple sources of information, including historical documents, oral histories and ethnographic data; sources not typically used in ecological studies [24,25]. Historical data, when available and properly analyzed, can yield a surprising wealth of information about species and ecosystems in the past, information that would be impossible to obtain using standard ecological research methods [4,26–29]. Oral histories provided by inhabitants of regions where commercial hunting took place can provide fundamental insights to our understandings of the impacts on and resilience of targeted species populations. Such historical methods can be applied to studies of contemporary management and conservation, contributing to the emerging field of “historical ecology” [30].

In this study, we draw on historical documents from regional port registries and shipping manifests as well as oral histories of local indigenous hunters to assess the impacts of the international fur trade on otter populations of the mid and upper Rio Negro, Amazonian Brazil. We reconstruct the overall harvest of the two otter species in the mid-upper Rio Negro throughout the twentieth century from both primary documents and oral histories. Finally, we assess the biological underpinnings of the differential resilience of the two species within the Rio Negro basin.

### Study area and cultural context

**Fig 1:**
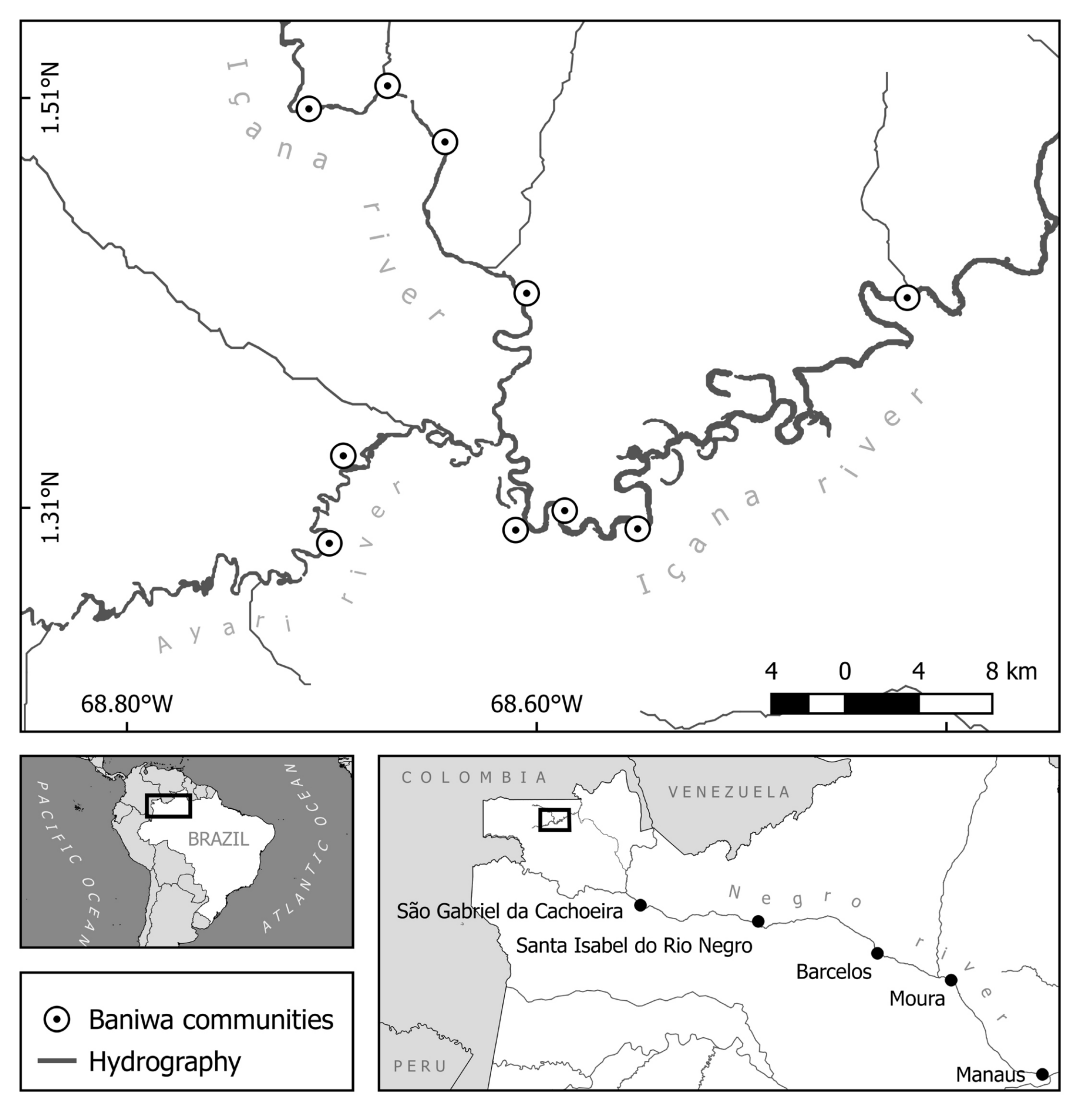
Study area. Location of Baniwa communities on the middle Rio Içana visited during this study (top), and twentieth century municipal centers along the Rio Negro, Amazonas, Brazil (bottom).

The mid-upper Rio Negro is located in the northwest Amazon within the Brazilian municipalities of Barcelos, Santa Izabel do Rio Negro and São Gabriel da Cachoeira (Fig 1), along the border with Colombia and Venezuela. The region is home to a tremendous diversity of indigenous peoples, including some twenty ethnic groups, speaking languages belonging to five distinctive cultural-linguistic families: Tukanoan, Arawakan, Maku, Yanomami and Tupi-Guarani [31]. Their traditional territory in Brazil is currently protected by five indigenous reserves totaling 10.6 million hectares, known collectively as the Upper Rio Negro Indigenous Lands [32]. The Baniwa, and their closely related neighbors in Colombia, the Coripaco, belong to the Arawakan language family and have inhabited the Içana river basin for centuries [33]. During the second half of the twentieth century, Baniwa hunters of the Rio Içana sold otter skins to the Manaus-based river trading empire of J.G. Araujo Ltda, and later to several other companies. Older Baniwa men who hunted during this time represent a valuable, but disappearing, source of information about this trade and its impact on otter populations.

In Brazil, the Içana basin is a black water river system dominated by low-productivity white-sand savannas. Local variations in water level, soil type and human habitation history create a diverse mosaic of landscapes and vegetation types [34,35]. The middle Içana, in the confluence with the Rio Ayari, is dominated by seasonally-flooded *igapó* forests on sandy soils. Though extremely poor in nutrients and mostly inappropriate for agriculture due to annual flooding, this region contains numerous blackwater lakes that are valued by local people for their relatively abundant fish populations [35]. Such lakes also provides good habitat for otters [13,36]. This region comprises the traditional territory of the *Dzawinai* or “jaguar people,” one of several patrilineal Baniwa clans.

The *Dzawinai*, once feared and respected for their bravery and shamanic prowess, were decimated by violence and exploitation by rubber merchants and Brazilian and Colombian military forces during and after the Rubber Boom [33]. Today, this “lakes region” as the Baniwa call it, covering some 65 km of river extension, is home to ten Baniwa communities from several different clans. We visited nine of these communities for this study: Jandu Cachoeira, Tucumã, Bela Vista, Urumutum Lago, São José do Ayari, Arapasso, Tucunaré Lago, Tarumã and Santa Marta.

## Methods

### Baniwa oral histories

To reconstruct the history of commercial hunting on the Rio Içana, we carried out semi-structured interviews [37] with all 11 residents, aged 59 to 88, from the nine study communities who were old enough to have participated in otter pelt hunting along the Içana. Research was carried out between September and November of 2015. All interviews were conducted in the company of our Baniwa co-author and research collaborator (Valêncio W. Macedo), who translated and otherwise facilitated communication and community relations (see S1 Appendix for details). Respondents were free to refuse participation in the research. All those who participated signed a formal Term of Free and Informed Consent. The study was approved by the Ethics Committee on Human Research of the National Institute of Amazonia Research (permit number 1.166.446) and by the National Research Ethics Committee (permit number 1.337.043). Ethnographic data concerning direct informant memories was supplemented with a review of anthropological and historical sources on the Baniwa and other indigenous groups of the upper Rio Negro, concerning their relationships with twentieth century traders.

### Commercial shipping records

**Fig 2:**
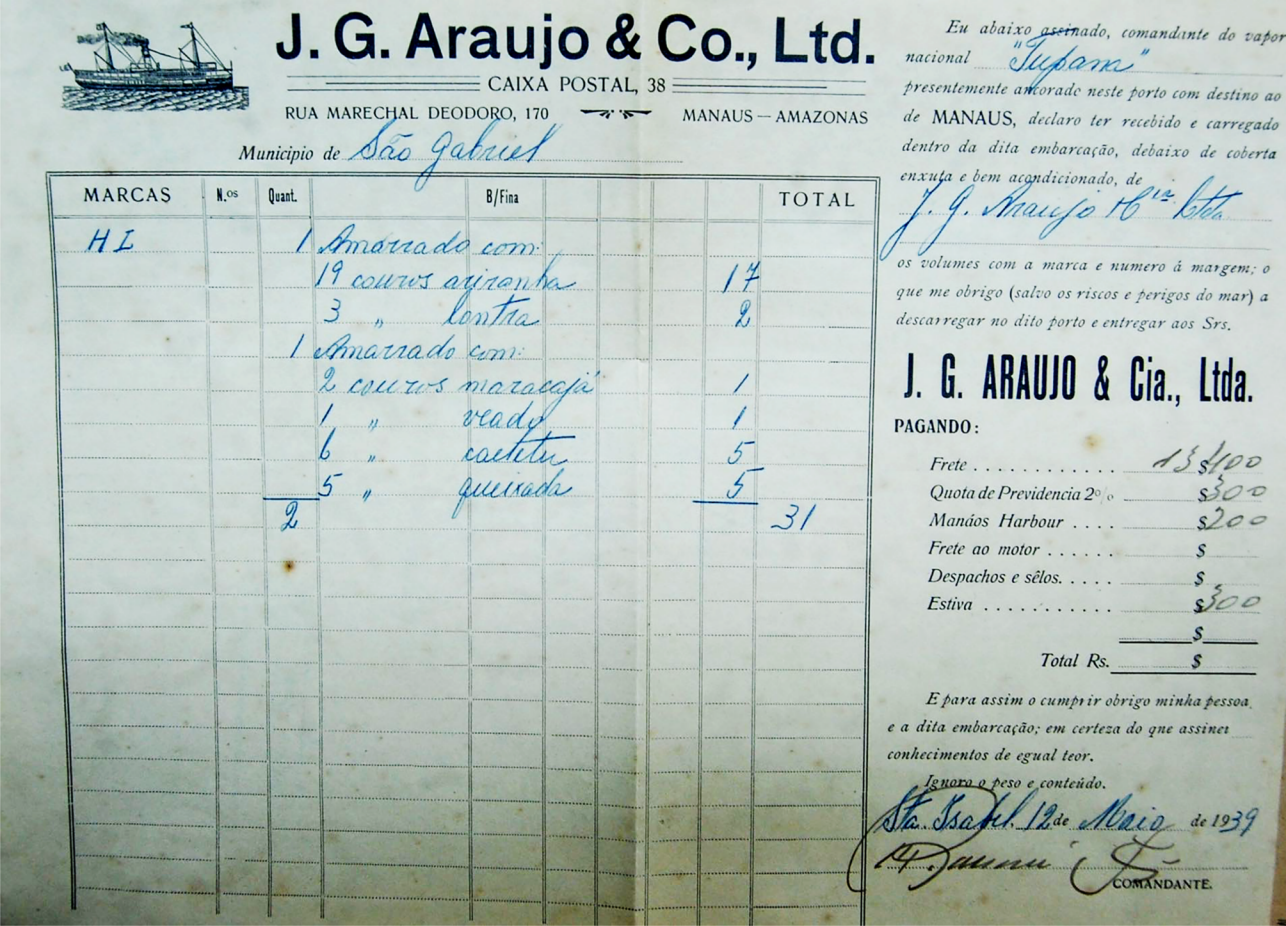
Bill of sale of hides transported by the boat “Tupana” from São Gabriel da Cachoeira (port of Santa Izabel do Rio Negro) to Manaus by the company J.G. Araujo Ltda. in 1939. The bill specifies one bundle containing nineteen hides of “ariranha” giant otter (*Pteronura brasiliensis*) and two hides of “lontra” neotropical otter (*Lontra longicaudis*); a second bundle containing two hides of “maracajá” generic commercial name for both ocelot (*Leopardus pardalis*) and margay (*L*. *wiedii*), one hide of “veado” red brocket deer (*Mazama americana*), six hides of “caititu” collared peccaries (*Pecari tajacu*) and five hides of “queixada” white-lipped peccaries (*Tayassu pecari*).

To assess the impact of commercial hunting on neotropical and giant otter populations of the Rio Negro, we analyzed bills of sale and cargo manifests (Fig 2) for boats and ships at the port of Manaus between 1936 and 1968. We had two sources of historical documents covering two distinct periods:

(A) 1936–1953:- shipping records, cargo manifests and financial documents of commercial boats owned by J.G. Araujo Ltda., now deposited in the Amazonian Museum of the Federal University of Amazonas State;
(B) 1958–1968: shipping records gleaned from cargo manifests of boats and ships from several trading companies based at the Port of Manaus, as published in the daily commercial shipping newspaper *Boletim Informativo Corel*. See [4] for further details about these historical documents.

**Fig 3:**
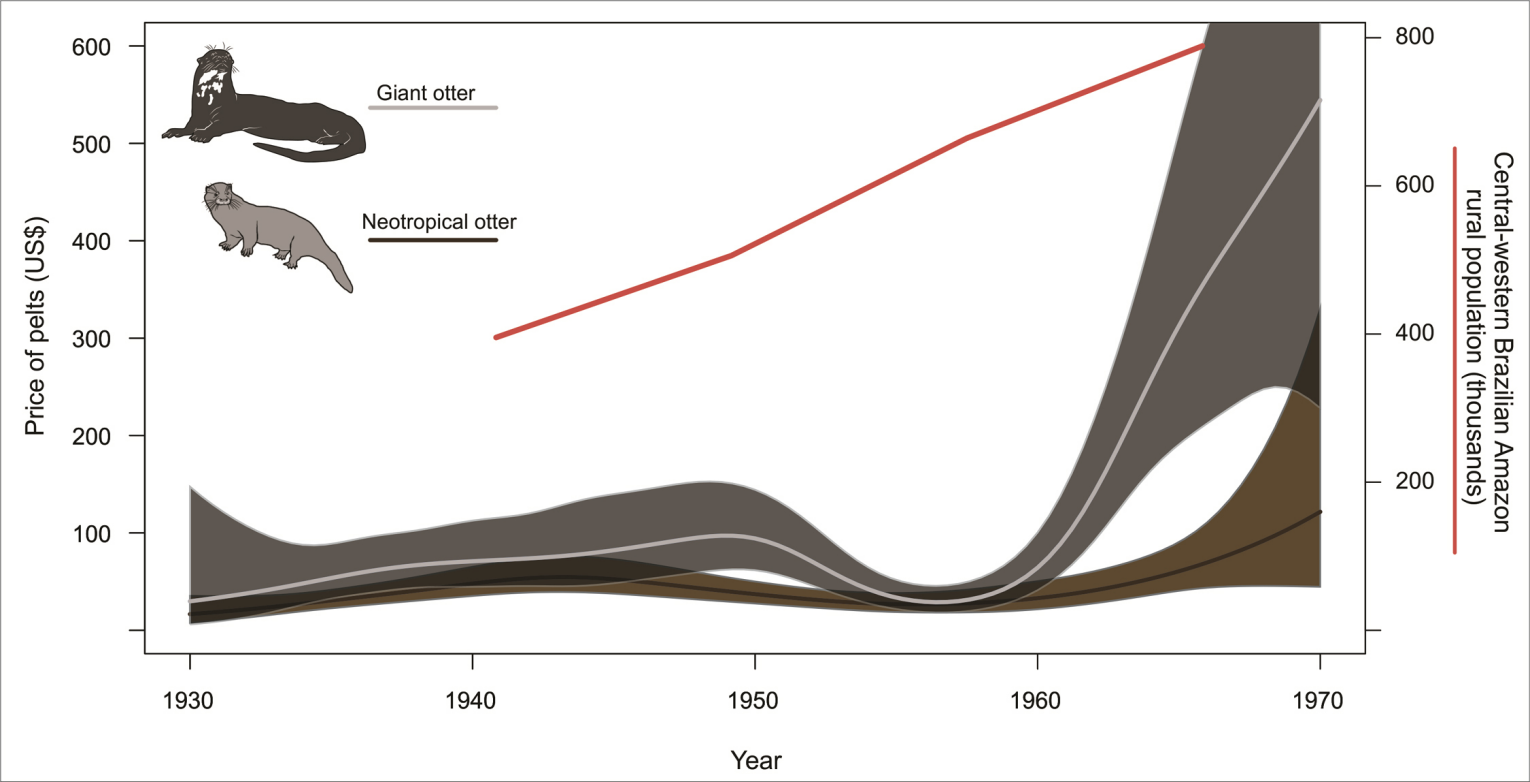
Hunting effort during the study period. Left side: price of otter pelts by species (note the quickly rising price for giant otter pelts during the 1960); right side: rural population in Amazonas State.

Since the structure of the two datasets is considerably different, we modeled the number of pelts traded per species separately for each time series. To capture temporal trends in the trade of otter pelts we modeled both time series using all Linear Models, Generalized Linear Models (GLM) and Generalized Additive Models (GAM). We then selected the best models by minimizing AIC (see S1 Table for details). To use our modeled harvest trend curves to draw inferences about population resilience during the hide trade, we compared the number of pelts traded at the beginning and end of both time series, during which periods hunting incentives were strong due to high market pelt prices (Fig 3). For species *i*, the estimated percentage change was given by:$100\left\{\sum \hat{\gamma_{i}}\;\left(last\;3\;years\right)-\;\sum \hat{\gamma_{i}}\;\left(first\;3\;years\right)/\sum \hat{\gamma_{i}}\;\left(first\;3\;years\right)\right\}$, where $\sum \hat{\gamma_{i}}$ denotes the sum of estimated harvests ${\hat{\gamma }}_{i}\left(t\right)$ over first or last three consecutive years. All analyses were performed in R (R Development Core Team) with the *mgcv* package for GAM [38]. Graphics were constructed using the *visreg* package [39].

Because of the unregulated and opportunistic nature of hunting practices in Amazonia, there is no direct information about hunting effort over time. However, there is strong secondary evidence to assume that hunting effort was increasing: both market prices and the rural human population in the central-western Brazilian Amazon surged during the 1960s (see historical demographic series on [40]), so it is reasonable to assume that hunting effort was also increasing during the study from 1940 to 1970 period. Hunting effort is unlikely to have decreased in response to declines in exploited populations because the wide range of commercially-attractive species ensured that hunters could trade whatever they could catch. Also, since animal skins constituted just one of many extractive products shipped by the fluvial transport network, the opportunity to sell hides persisted even when the volume of trade diminished. It is therefore reasonable to assume that harvest trends reflected animal population status to some degree, especially in the case where low harvests were registered despite strong market incentives and a high human population. Complete raw data from shipping records is present in S2 and S3 Tables.

## Results

### History of commercial hunting on the upper Rio Negro

During the initial Rubber Boom at the turn of the 20^th^ century, commerce on the Rio Negro was controlled by J.G. Araujo [41]. According to the travelogues of ethnologist Gordon MacCreagh, who visited the region in 1926, “J.G.” (as Sr. Araujo was known) warned him not to visit the Rio Içana, noting both the difficulty of its access and the hostility of the Baniwa inhabitants toward non-indigenous people [41]. The Baniwa’s fierce reputation at the time stemmed from their violent rebellion against rubber bosses and *regatões* (merchant ship owners) during the late 19^th^ century [33]. Despite these warnings, MacCreagh visited the Baniwa and described them as friendly, but noted interethnic violence resulting from competition between the “King of the Içana”, a Spanish merchant who controlled commerce along this river, and the “King of the Vaupes,” a Portuguese colonist who controlled the neighboring river’s commerce and was notorious as "the man who makes the water bloody" (see p. 251–274 in ref. [41]).

According to Baniwa oral histories, some local commercial activities on the Içana began with the establishment of a Salesian mission on the lower Içana in the 1920s. Commercial hunting was further incentivized during the 1940s with the arrival of Sophia Muller, an American Protestant missionary of the New Tribes Mission who evangelized some Baniwa clans and the neighboring Coripaco [33]. The combined presence of Catholic missionaries on the lower Içana and Protestant missionaries on the middle and upper Içana brought an end to the reign of the warring “Kings” described by MacCreagh, and opened the Içana to outside traders.

Prior to World War II, commercial trade on the upper Rio Negro involved diverse forest products such as *breu* resin (*Protium* spp., Burseraceae), *sorva* (*Couma* spp., Apocynaceae), Brazil nuts (*Bertholethia excelsa*, Lecythidaceae) and bushmeat, as well as native crafts like manioc graters and traditional basketry. Around 1945, according to our informants, a group of Baniwa men migrated to the towns of Santa Izabel and Barcelos on the middle Rio Negro to harvest Brazil nuts and extract rubber. As a result, traders there became aware of the commercial potential that existed farther up river. Two of our interview participants mentioned isolated cases of pelt hunting on the Içana in the 1920s and 1940s (Table 1), but it was only around 1950 that commercial pelt hunting became truly common on the Içana. By the early 1960s, pelt hunting was the main commercial activity in the region.

**Table 1:** Baniwa oral histories of commercial hunting activity on the middle Içana during the 20th century international fur trade.

Age	Baniwa’s reports	Start	Decline	End reasons
84	“The giant otter hunting began when I was still living on Pamáali creek, just before I got married. I must have been about 20 at the time.”	± 1951	1975	Giant otter extinction
88	“I was very young then the whites began the hunting here. I was 14 when I went after giant otter for the first time.”	± 1940	1948	Giant otter extinction
71	“I was very small when the hunting started here, but I remember my dad and granddad going hunting. They would tell me how they hunted, and said that the whites began to appear around about 1950.”	± 1950	> 1960	1967’s law and giant otter extinction
62	“Myself, I didn’t actually hunt. My father said that in the time before he got married many people were taken to work in Barcelos. That stopped around 1950, which was when the hunting and the trading started here.”	± 1950	> 1960	Giant otter extinction
59	“I don’t remember the dates, but I was already grown up when the river traders arrived here in Ayari looking for skins. I was already fishing at the time. I was about 12 when they first appeared.”	± 1966	1969	Giant otter extinction
66	“It was when Sophie Muller arrived that the white traders arrived and started trading on the Rio Içana. However, hunting of giant otters only happened afterwards. Those traders came about 10 years later”	± 1954	< 1960	Giant otter extinction
62	“I’m not really sure when the hunting started here. But by the time I was 14, there were already white traders coming to the communities looking to buy skins. We swapped the skins for salt, sugar, soap …”	< 1967	± 1972	Giant otter extinction
62	“My father said that when he and his brothers founded this community, there were already traders in the region looking for skins of giant otter, river otter, jaguar. . .”	1925	> 1960	Giant otter extinction
59	“My grandfather said that when he arrived in the community (1925) there were already river traders in the region, but there were few. It was in Juscelino’s time that the trade in skins really began.”	± 1956	> 1970	Giant otter extinction
52	“When I was born, there were no longer any giant otters in the region. But my dad told me that he would go and hunt up on Pamáali Creek before moving here.”	< 1955	1963	Giant otter extinction
63	“I must have been less than 10, but I remember the Içana full of white traders’ boats, in search of skins of jaguar, margay, river otter and giant otter. The trade lasted nearly 10 years, until the animals disappeared from around here. No-one saw giant otters again.”	> 1955	> 1960	Giant otter extinction

“During this time there were lots of giant otters, everywhere. You didn’t have to go far to hunt them, no indeed”.

Pedro Brazão (71 years old, Tucumã community)

By the early 1960s, trading boats from São Gabriel and Manaus had become a common sight along the Içana. They would stop at Baniwa settlements to purchase or place “orders” for luxury pelts of both otter species, as well as felids like jaguar (*Panthera onca*), ocelot (*Leopardus pardalis*), and margay (*Leopardus wiedii*). Giant otter and jaguar pelts were the most profitable. A top quality giant otter pelt could purchase up to three new rifles while a neotropical otter pelt was worth only one rifle. Although a jaguar skin had about the same value as that of a giant otter, jaguar hunting was more difficult and dangerous when compared with otters, which could be encountered opportunistically on fishing expeditions.

**Fig 4:**
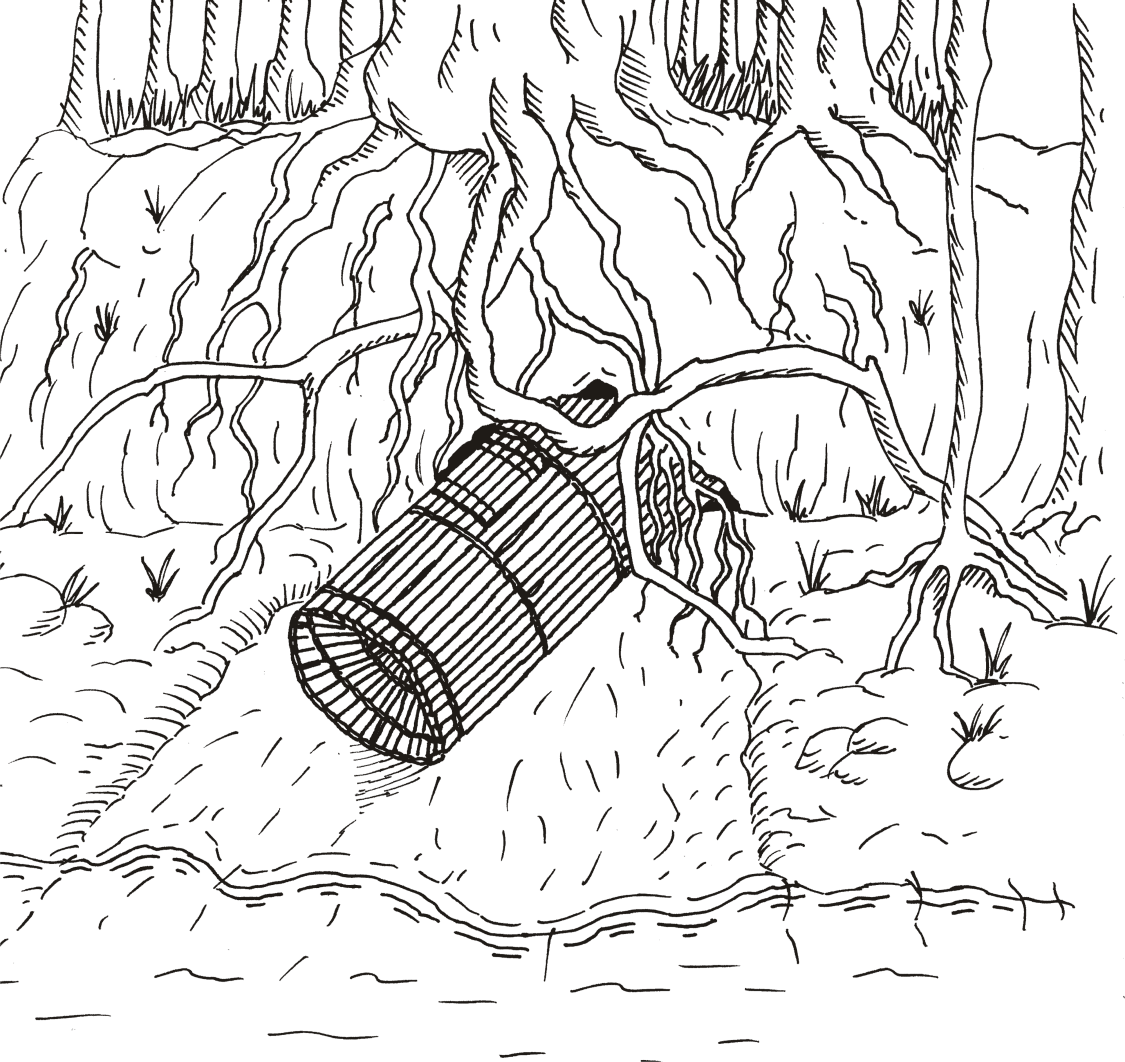
Hunting with matapi fishing trap. During the heyday of the 20^th^ century fur trade, Baniwa hunters captured giant otters by placing the *matapi* trap at the entrance to the den (Illustration by Ramiro Melinski).

At that time, otters were found all along the Içana River, in large lakes and on tributary streams. In the lakes region, hunters typically conducted hunting expeditions lasting 15–20 days, and would target entire giant otter social groups. The neotropical otter was not initially targeted because of its lower value, but individuals were sometimes killed opportunistically when encountered. At first, the Baniwa hunted with bow-and-arrow or used a cone-shaped trap called *dzaarokana* in Baniwa (*matapi* in Portuguese) made from *Mauritia flexuosa* palm fibers, traditionally used to catch fish in river rapids [42]. They would follow giant otters groups all day in order to locate the den. When using bows and arrows, or later rifles, hunters would camp out and wait until sunrise, killing as many of the group as possible when they emerged. When using the *matapi*, they placed the trap at the den entrance at nightfall, trapping the entire group when it emerged the next morning (Fig 4). Traps were very efficient and ensured higher quality pelts (and higher prices) without arrow holes. However, cartridges and gunpowder became the main form of payment for pelts, replacing traditional hunting techniques with firearms and probably increasing hunting success [20].

“We stopped hunting because there were no more giant otters to hunt. They fled to the stream headwaters where no hunters could get them… We continued hunting neotropical otters, there were still plenty of those.”

Lúcio Pereira Paiva (59 years-old, Arapasso community).

**Fig 5:**
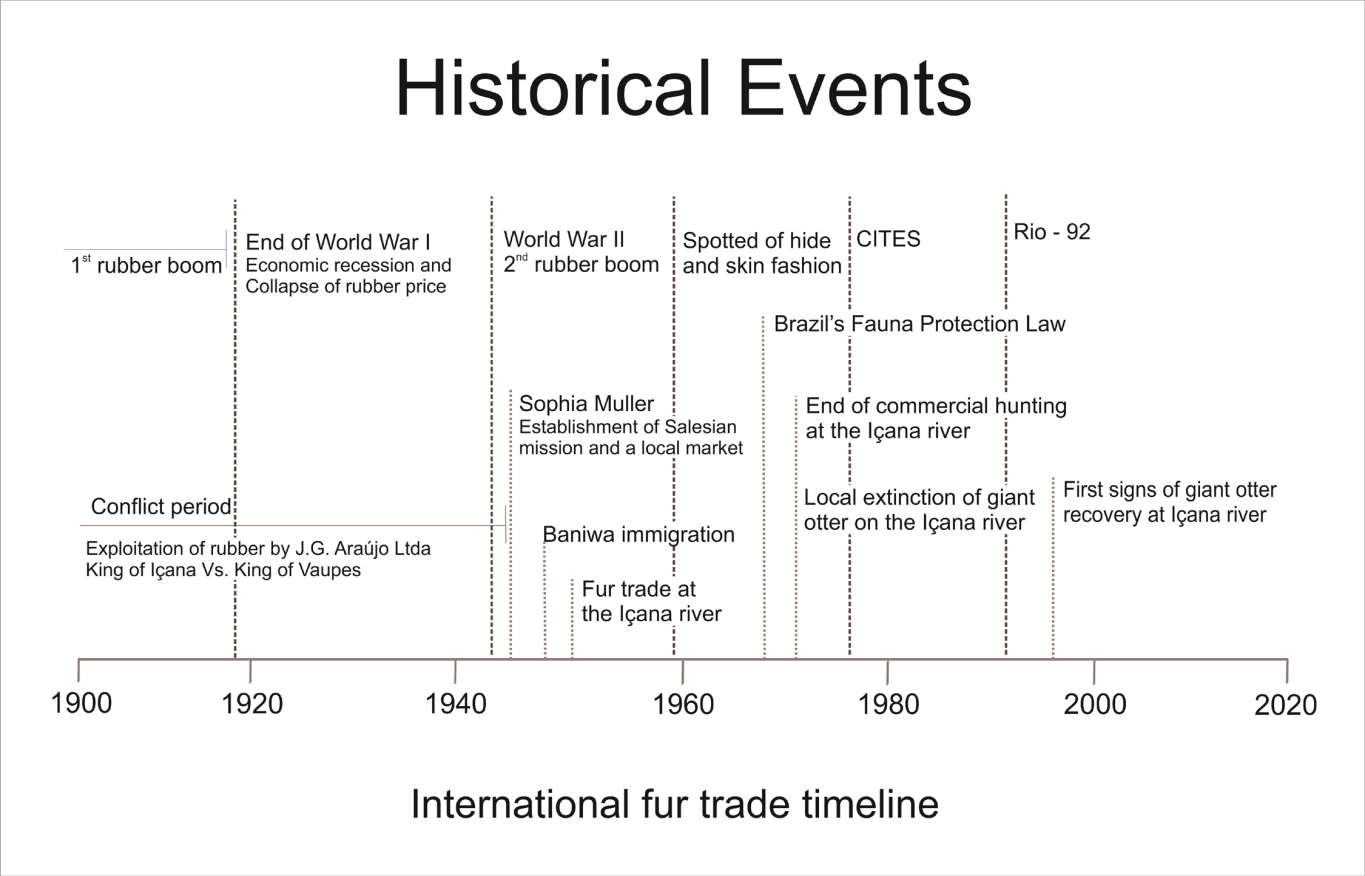
Timeline of historical events. National, international and regional historical events reconstructed based on ethnographic information and Baniwa oral histories concerning the commercial hunting for the international fur trade in the late 20^th^ century.

The hunting boom for the Baniwa was short-lived. According to oral histories, in the middle 1960s giant otter populations along the Içana began to decline precipitously. By the early 1970s there were no more giant otters to be found on the middle Içana. Apparently, surviving giant otter groups moved to remote stream headwaters, which became refuge areas for the species [36]. Neotropical otters persisted at lower densities than before the hunting boom. However, lower prices, and the ultimate collapse of the fur trade, made the species less attractive for commercial harvest. For a summary timeline of these historical events, see Fig 5.

“We Baniwa didn’t hunt the otters. We don’t eat these animals. It was the white people who told us to hunt them to take their skins. Once the white people left no one here ever hunted them again, and that’s why they’re coming back now.”

Alberto (84 years-old, Jandu Cachoeira community)

### Harvest trends in the otter fur trade on the Rio Negro

**Fig 6:**
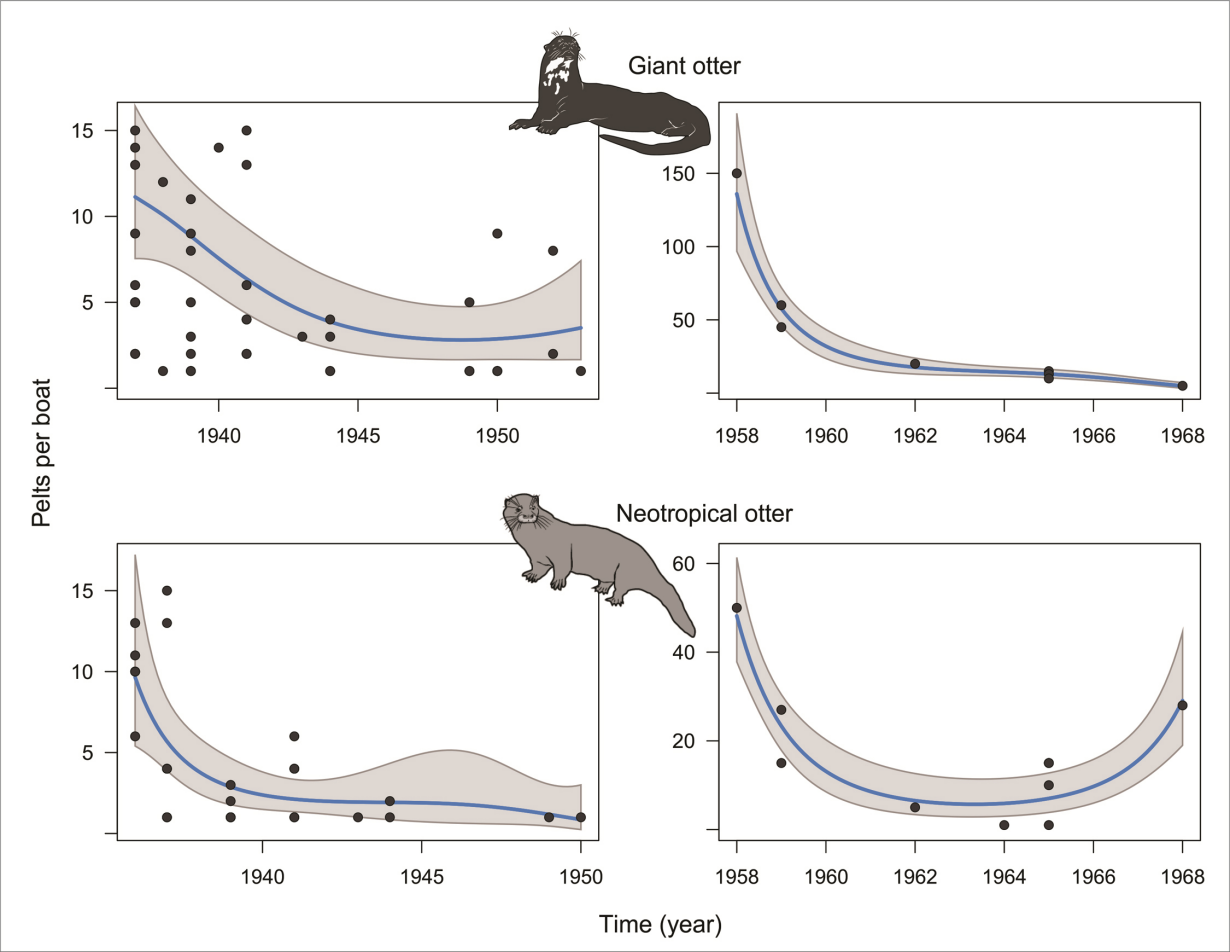
Number of giant otter and neotropical otter pelts landed at Manaus port by boats coming from the Rio Negro between 1935 and 1968. Time series on the left (from late 1930s to early 1950s) represent pelts traded by J.G. Araujo Company and time series on the right (from 1958 to 1968) are pelts traded by various other companies.

Our analysis of historical records of otter pelts landed at the port of Manaus between 1936 and 1968 from the Rio Negro (Fig 6) confirms the direct testimonies of Baniwa hunters concerning the differential impacts of commercial hunting on two species of Amazonian otters. From 1936 to 1953 there was a decline of 64% in the number of giant otter pelts and 76% in neotropical otter pelts traded by J.G. Araujo’s commercial fleet (Table 2), despite continuing demand and high prices. Data gleaned from the port newspaper from 1958 to 1968 show a severe decline of 90% in the number of giant otter pelts landed. However, despite an overall decline of 39% in neotropical otter pelts in the second time series (Table 2), there was a significant increase in the number of pelts of this species in the mid-1960s, coinciding with a second peak in international demand from the fashion industry [2]. We presume that the less valuable and more elusive neotropical otter became the target species as giant otter populations were driven extinct in accessible localities, as was previously reported in a 1985 investigation of the continuing illegal in pelts in Brazil despite the legal prohibition [43].

**Table 2:** Estimated percentage harvest change showing the change in modeled harvest for each species between the initial 3-year period and the final 3-year period of exploitation for the two time series.

**Giant otter** *Pteronura brasiliensis*						
1937–1953 (JG Araujo time series)			1958–1968 (Corel time series)			
Annual average harvest per boat		Percentage harvest change -64%	Annual average harvest per boat			Percentage harvest change -90%
1937–1939	1951–1953		1958–1960	1966–1968		
11	4		76	9		
**Neotropical otter** *Lontra longicaudis*						
1936–1950 (JG Araujo time series)			1958–1968 (Corel time series)			
Annual average harvest per boat		Percentage harvest change -76%	Annual average harvest per boat			Percentage harvest change -39%
1936–1938	1948–1950		1958–1960	1966–1968		
7	2		29	18		

**Fig 7:**
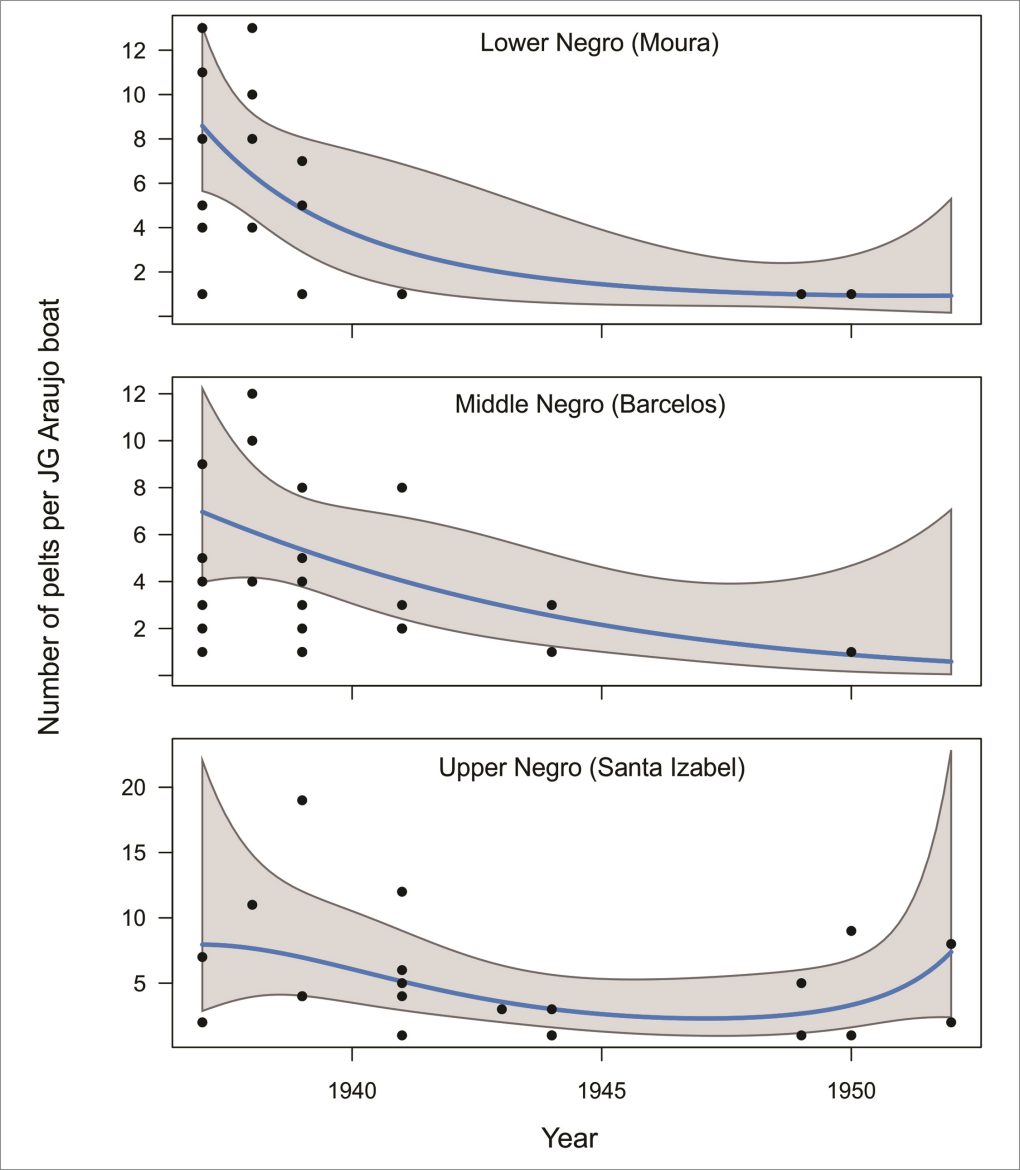
Number of giant otter skins landed at Manaus port between 1935 and 1953 from the municipalities of Moura, Barcelos and Santa Izabel. Data from cargo manifests of J.G. Araujo Company. Note that Sta. Izabel is the port located highest up along the Negro basin (see Fig 1).

Although otter landings from the lower and middle Negro decrease overall from 1936–1968, the port of Sta. Izabel, the main center of commerce for the upper Rio Negro at the time, shows a slight increase in giant otter pelt landings during the 1950s (Fig 7), though still attaining less than half of peak production in 1939. Baniwa oral histories likewise indicate an intensifying trade in otter pelts along the Içana during this period. As giant otter populations elsewhere apparently collapsed, traders sought out ever more remote areas to find them.

Giant otter pelt harvests already showed a dramatic decline in 1945. However, the harvest of neotropical otter pelts continued to increase until the end of the 1960s when the Fauna Protection Law of 1967 was passed. Moreover, the rural human population in the central-western Brazilian Amazon was 68% larger in the 1960s than in the 1940s [40]. Thus, species that disappeared from the harvest profile in the later period had presumably experienced widespread population decline along the Rio Negro. Although it cannot be proven that these harvest changes were due solely to overhunting, the circumstantial evidence is strong, especially when considered alongside oral histories from elder Baniwa hunters. Conversely, a greater resilience to exploitation can be deduced for the neotropical otter, for which harvests remained buoyant in the 1960s. Both oral history and data from historical documents indicate that ten years of commercial hunting was enough to cause the collapse of giant otter populations from the Rio Negro at local and regional levels. Despite the evident collapse of populations of giant otter, neotropical otter populations appear to have persisted at both scales.

## Discussion

### Differential resilience of otter species to commercial hunting in the twentieth century

The higher vulnerability of the giant otter, as compared to the neotropical otter, to commercial hunting [4] was supported both by oral histories of Baniwa people and by analysis of historical documents of otter pelt landings from the Negro basin. Even though they are from the same Mustelid sub-family (Lutrinae) [44], are sympatric and share a non- competitive mainly fish-based diet [45], neotropical and giant otters show important differences in reproductive strategy and social organization that result in divergent responses to hunting pressure.

Both species have gestation periods of between 50 and 70 days, can produce 1 to 5 offspring per year and are sexually mature at around two years of age [12,46,47]. However, giant otter females rarely reproduce before the age of three, due to hierarchical social behavior [48]. Moreover, neotropical otters are polygynous and dimorphic, with males being up to 25% larger than females [46]. This means that males and females can mate with multiple reproductive partners present in their territory, and that, to achieve maximum cost-benefit, Bawina hunting was probably male-focused. Since the loss of males, or even reproductively-active individuals, does not necessarily impact the entire local population, these biological and ecological characteristics contributed to the resilience of the neotropical otter to commercial hunting. In contrast, giant otters are monogamous, with only one active breeding pair per social group [12]. The loss of one member of the alpha breeding pair might easily fragment a group established in a given territory.

Neotropical otters are also smaller (5–15 kg), solitary, quiet, timid and although typically diurnal, can switch to a nocturnal habit when subjected to human pressure [46]. The neotropical otter can also inhabit smaller streams than the giant otter, which is much larger (22–29 kg) [49,50], diurnal, curious, highly social, and lives in groups of up to 20 individuals [12,48] that communicate conspicuously with a wide vocal repertoire [51]. These factors make the giant otter easier to detect and hunt in large numbers. Accounts of Baniwa hunting strategies clearly reflect these differences between the two species.

High prices for giant otter pelts also resulted in greater hunting pressure, confirming the role of local depletion, price appreciation and expanding spatial scale of commercial hunting [52] that also contributed to the regional depletion of feline species [8]. Such features of commercial hunting contrast with the more flexible central-place foraging strategies of subsistence hunters [15,19,20,22,23]. Moreover, ease of access to riverine habitats [53], when compared to terrestrial habitats, served to increase the impact of hunting on high-value aquatic species, such as otters, manatee, caiman and capybara, during the heyday of commercial hunting in the Brazilian Amazon [4,10] (See Table 3 for a summary of biological, cultural and economic aspects influencing the differential resilience of otters in aftermath the international fur trade).

**Table 3:** Elements of vulnerability. Summary of biological, ecological, economic and cultural aspects of hunting that may have influenced the differential resilience of otters in the aftermath of the commercial hunting for the 20^th^ century fur trade.

Elements of Vulnerability	Neotropical Otter	Giant Otter
***Body size***	Small-sized[49]	Large-sized[49]
***Sexual dimorphism***	Dimorphic[46]	Not dimorphic[50]
***Sexual behavior***	Polygynous[44,46]	Monogamous[12,44]
***First offspring***	2 years[46]	2–3 years[47, 48]
***Annual offspring***	0,575[44]	0,575[12,44]
***Social strategy***	Solitary[44,46]	Social[12,44,48]
***Activity pattern***	Mainly diurnal[44,46]	Diurnal[12,44,48]
***Animal behavior***	Inconspicuous[44,46]	Conspicuous[44,51]
***Maximum intrinsic rate***	Rmax = 0,32[4]	Rmax = 0,26[4]
***Price appreciation***	Medium-value pelt[2,4]	High-value pelt[2,4]
***Hunting strategy***	Opportunistic	Directed
***Hunting success***	Sporadic	Easy to kill
***Spatial hunting pattern***	Restrict to fishing areas	Widespread to expeditions areas

Interpreting oral histories of Baniwa hunters in the light of historical data, the progressive extinction of giant otter populations along the lower and middle Negro led traders to seek out the remote Içana basin to continue supplying international markets with furs, especially as demand peaked again in the 1960s. Yet by the time the Brazilian Fauna Protection Law was passed in 1967, commercial hunting had already driven giant otter populations to local extinction in all accessible regions of the Içana basin. As giant otter populations dwindled, pelts of the less-valued, but more resilient, neotropical otter entered the market as a substitute product. A similar pattern was observed for two species of Amazonian caiman, where the more-valued black caiman skins were eventually replaced in the international trade by those from spectacled caiman when the former species was driven to local extinction in the mid-20^th^ [4].

From a historical perspective, the power of the international market to cause a collapse of animal populations has been observed around the world. Innovations in tanning technology in Europe and robust demand for hides, stable or rising prices and unregulated exploitation wiped out the American bison (*Bison bison*) in the late 19th century [54]. Between 10 and 15 million bison were slaughtered in a little over 10 years for their hides (1870s), with only 100 individuals remaining in the Great Plains states in the late 1880s [54].

Far from an exclusively historical issue, the massive demand for wildlife products for international trade continues to cause severe wildlife population declines in elephants, rhinos and tigers in Africa and Asia [55]. With the arrival of Chinese investors to the Amazon in recent years, an illegal international trade in fangs, claws and fur of jaguars and other felids for Asian markets has reemerged [56,57]. As Asian tiger populations have become alarmingly reduced, Chinese traders seek jaguar parts as substitute products. Despite national and international bans on such trade, poor patrolling ability and governance in the Amazon region, the inherent vulnerability of large carnivores to hunting and habitat destruction may lead to new wildlife population collapses in the 21^st^ century.

### Population recovery: Implications for mustelid conservation

Currently, neotropical otter populations appear to have recovered to historical levels throughout the species’ distribution [58]. However, *Lontra longicaudis* continues to be classified as "Near Threatened" by the IUCN Red List due to predicted anthropogenic impacts in their habitat [59]. The giant otter was driven to local extinction in many places throughout the Amazon. However, with the ban on commercial hunting and the decrease in market demand, populations appear to be recovering in much of its historical range in Colombia [60], Peru [9], the Brazilian Amazon and Pantanal [61,62], including the upper Rio Negro [36]. Nevertheless, *Pteronura brasiliensis* remains as "Endangered" on the IUCN Red List, since human actions in their habitat continue to threaten population recovery [63].

Population recovery for both species is linked to Brazil’s 1967 Fauna Protection Law and especially to the country’s ratification of CITES in 1975 [9]. However, population recovery requires the maintenance of refuge areas that are sufficiently remote as to be mostly free of human pressure, and with populations close to carrying capacity [64] that are capable of recolonizing nearby depleted areas via source-sink dynamics [65,66]. Indeed, the Baniwa recognize such source-sink dynamics in action, as they attribute the return of the giant otter, which they refer to as the “shaman of the waters”, to colonization from remote headwater refuges [36].

Human-inhabited indigenous and extractive reserves, as well as more strictly protected conservation areas, provide important source areas for the protection and recolonization of threatened and endangered species. The presence of remote areas within protected Indigenous Lands apparently guaranteed the survival of refuge populations, allowing both species of Amazonian otter to persist and recover. This study emphasizes the potential contributions of historical analysis and ethnography to interdisciplinary research on ecological processes, while also highlighting the role of community participation in applied research and management of protected areas.

## Supporting information

S1 Table Result of time series modeling using Linear Models, Generalized Linear Models and Generalized Additive Models, and the respective minimizing AIC. (DOCX)

S2 Table Number of neotropical otter and giant otter skins listed per boat per year between 1937 and 1953 at the Port of Manaus by the JG Araujo company. The data is now deposited in the Amazonian Museum of the Federal University of Amazonas State, Brazil. (DOCX)

S3 Table Number of neotropical otter and giant otter skins listed per boat per year between 1957 and 1968 at the Port of Manaus. The data was obtained from a Manaus mercantile newspaper, the *Boletim Informativo Corel*. (DOCX)

S1 Appendix Explanatory guide to the Baniwa oral histories of otter hunting and the 20th century fur trade. (DOCX)
