# Supplementary material for: Differential resilience of Amazonian otters along the Rio Negro in the aftermath of the 20th century international fur trade
Source: PLoS One. 2018 Mar 30;13(3):e0193984. doi: 10.1371/journal.pone.0193984 (PMC5877832; doi:10.1371/journal.pone.0193984)
Supplement: S1 Table — (DOCX) [file pone.0193984.s001.docx]

**Table S1 Result of time series modeling using Linear Models, Generalized Linear Models and Generalized Additive Models, and the respective minimizing AIC.**

| Model | Giant otter | | Neotropical otter | |
| --- | --- | --- | --- | --- |
|  | 1937-1953 | 1958-1968 | 1936-1950 | 1958-1968 |
|  | AIC values | | | |
| Null | 360.8 | 87.7 | 160.1 | 85.5 |
| Linear | 354.7 | 82.2 | 152.9 | 85.3 |
| GLM (Poisson) | 466.6 | 83.7 | 140.5 | 143.3 |
| GLM (Gamma) | 303.8 | 59.1 | 125.7 | 81.4 |
| GLM Polynomial 2º order (Gamma) | 304.8 | 59.8 | 124.6 | 77.1 |
| GLM Polynomial 3º order (Gamma) | 304.9 | **52.0** | **124.5** | 78.4 |
| GLM Polynomial 2º order (Gaussian) | 355.9 | 64.3 | 146.0 | 69.9 |
| GLM Polynomial 3º order (Gaussian) | 356.1 | 55.9 | 147.4 | **70.4** |
| GAM (Gaussian) | **303.5** | - | - | - |
| GAM (Gamma) | 355.3 | - | - | - |
