# Supplementary material for: Differential resilience of Amazonian otters along the Rio Negro in the aftermath of the 20th century international fur trade
Source: PLoS One. 2018 Mar 30;13(3):e0193984. doi: 10.1371/journal.pone.0193984 (PMC5877832; doi:10.1371/journal.pone.0193984)
Supplement: S2 Table — (DOCX) [file pone.0193984.s002.docx]

**S2 Table Number of neotropical otter and giant otter skins listed per boat per year between 1937 and 1953 at the Port of Manaus by the JG Araujo company. The data is now deposited in the Amazonian Museum of the Federal University of Amazonas State, Brazil.**

|  | JG Araujo time serie | | | |
| --- | --- | --- | --- | --- |
|  | ***Giant otter*** | | ***Neotropical otter*** | |
| Boat nº | Year | Amount of skin | Year | Amount of skin |
| 1 | 25 | 1937 | 13 | 1936 |
| 2 | 2 | 1937 | 11 | 1936 |
| 3 | 13 | 1937 | 1 | 1936 |
| 4 | 5 | 1937 | 1 | 1936 |
| 5 | 5 | 1937 | 6 | 1936 |
| 6 | 5 | 1937 | 15 | 1937 |
| 7 | 5 | 1937 | 4 | 1937 |
| 8 | 9 | 1937 | 1 | 1937 |
| 9 | 2 | 1937 | 4 | 1937 |
| 10 | 6 | 1937 | 4 | 1937 |
| 11 | 14 | 1937 | 1 | 1937 |
| 12 | 15 | 1937 | 13 | 1937 |
| 13 | 27 | 1938 | 2 | 1939 |
| 14 | 12 | 1938 | 1 | 1939 |
| 15 | 1 | 1938 | 3 | 1939 |
| 16 | 23 | 1938 | 1 | 1939 |
| 17 | 2 | 1938 | 1 | 1939 |
| 18 | 3 | 1938 | 6 | 1941 |
| 19 | 11 | 1939 | 1 | 1941 |
| 20 | 3 | 1939 | 4 | 1941 |
| 21 | 8 | 1939 | 4 | 1941 |
| 22 | 31 | 1939 | 1 | 1941 |
| 23 | 5 | 1939 | 1 | 1943 |
| 24 | 1 | 1939 | 2 | 1944 |
| 25 | 1 | 1939 | 1 | 1944 |
| 26 | 1 | 1939 | 1 | 1949 |
| 27 | 9 | 1939 | 1 | 1950 |
| 28 | 2 | 1939 |  |  |
| 29 | 14 | 1940 |  |  |
| 30 | 4 | 1941 |  |  |
| 31 | 2 | 1941 |  |  |
| 32 | 4 | 1941 |  |  |
| 33 | 15 | 1941 |  |  |
| 34 | 13 | 1941 |  |  |
| 35 | 2 | 1941 |  |  |
| 36 | 6 | 1941 |  |  |
| 37 | 3 | 1943 |  |  |
| 38 | 1 | 1944 |  |  |
| 39 | 1 | 1944 |  |  |
| 40 | 4 | 1944 |  |  |
| 41 | 3 | 1944 |  |  |
| 42 | 5 | 1949 |  |  |
| 43 | 1 | 1949 |  |  |
| 44 | 1 | 1949 |  |  |
| 45 | 1 | 1950 |  |  |
| 46 | 9 | 1950 |  |  |
| 47 | 1 | 1950 |  |  |
| 48 | 1 | 1950 |  |  |
| 49 | 8 | 1952 |  |  |
| 50 | 2 | 1952 |  |  |
| 51 | 1 | 1953 |  |  |
