# Supplementary material for: Differential resilience of Amazonian otters along the Rio Negro in the aftermath of the 20th century international fur trade
Source: PLoS One. 2018 Mar 30;13(3):e0193984. doi: 10.1371/journal.pone.0193984 (PMC5877832; doi:10.1371/journal.pone.0193984)
Supplement: S3 Table — (DOCX) [file pone.0193984.s003.docx]

**S3 Table Number of neotropical otter and giant otter skins listed per boat per year between 1957 and 1968 at the Port of Manaus. The data was obtained from a Manaus mercantile newspaper, the *Boletim Informativo Corel.***

|  | *Corel time serie* | | | |
| --- | --- | --- | --- | --- |
|  | ***Neotropical otter*** | | ***Giant otter*** | |
| Boat nº | Year | Amount of skin | Year | Amount of skin |
| 1 | 1957 | 2 | 1957 | 2 |
| 2 | 1958 | 150 | 1958 | 150 |
| 3 | 1959 | 60 | 1959 | 60 |
| 4 | 1959 | 45 | 1959 | 45 |
| 5 | 1962 | 20 | 1962 | 20 |
| 6 | 1965 | 15 | 1965 | 15 |
| 7 | 1965 | 13 | 1965 | 13 |
| 8 | 1965 | 10 | 1965 | 10 |
| 9 | 1968 | 5 | 1968 | 5 |
