# Supplementary material for: Differential resilience of Amazonian otters along the Rio Negro in the aftermath of the 20th century international fur trade
Source: PLoS One. 2018 Mar 30;13(3):e0193984. doi: 10.1371/journal.pone.0193984 (PMC5877832; doi:10.1371/journal.pone.0193984)
Supplement: S1 Appendix — (DOCX) [file pone.0193984.s004.docx]

**Interviwe Guide**

| **Respondent:** | |
| --- | --- |
| **Date:** | **Community:** |
| **Age:** | **Ethnic group:** |

**Item 1- History of the respondent**

| - Where you were born? |
| --- |
| - How long have you lived in this community? |
| - What is your main activity here? |

**Item 2- History of commercial hunting of otters**

| - Is there any kind of otter in the Içana river? | | |
| --- | --- | --- |
| If yes: | Please, tell me about them/it. | |
| - Do you know if someone have ever hunted otter here? | |  |
| If yes: | When they start hunting otter on the Içana river?  When did they usually hunted them (season)?  What were the otter hunting tecnniques?  Why did they hunt otters?  Who usually bought the pelts?  How much did they cost? |  |
| - Do anybody hunt otters nowadays? | |  |
| If yes: | What for? |  |
| If no: | When did the Baniwa stop hunting otter? Why? |  |

**Item 3- Otter populaton trends and Baiwa perceptions about otters**

| - Have these two species always lived here? | |
| --- | --- |
| If no: | When did they disappear?  Why do you think they declined/disappeared?  When they disappeared from around here, were there some areas where you could still find them?  Have you noticed any changes in lakes/creeks since they disappeared? |
| - Do you think they are back now? | |
| If yes: | When they start to recover?  Do you remember for each lake/stream when they started to come back?  Have you noticed any changes in the lakes/creeks since they came back? |
| - What do you think about the return of otters to the Baniwa territory? | |
| - Do you know any myths about otters? Could you tell me? | |
